# Revealing genes associated with vitellogenesis in the liver of the zebrafish (*Danio rerio*) by transcriptome profiling

**DOI:** 10.1186/1471-2164-10-141

**Published:** 2009-03-31

**Authors:** Liraz Levi, Irena Pekarski, Ellen Gutman, Paolo Fortina, Terry Hyslop, Jakob Biran, Berta Levavi-Sivan, Esther Lubzens

**Affiliations:** 1 Department Marine Biology and Biotechnology, Israel Oceanographic and Limnological Research, Haifa, Israel; 2 Department of Animal Sciences, Faculty of Agricultural, Food and Environmental Quality Sciences, The Hebrew University of Jerusalem, Rehovot 76100, Israel; 3 Department of Cancer Biology, Kimmel Cancer Center, Thomas Jefferson University Jefferson Medical College, Philadelphia, PA 19107, USA; 4 Department of Experimental Medicine, University La Sapienza, Roma, Italy; 5 Division of Biostatistics, Thomas Jefferson University Jefferson Medical College, PA 19107, USA

## Abstract

**Background:**

In oviparous vertebrates, including fish, vitellogenesis consists of highly regulated pathways involving 17β-estradiol (E2). Previous studies focused on a relatively small number of hepatic expressed genes during vitellogenesis. This study aims to identify hepatic genes involved in vitellogenesis and regulated by E2, by using zebrafish microarray gene expression profiling, and to provide information on functional distinctive genes expressed in the liver of a vitellogenic female, using zebrafish as a model fish.

**Results:**

Genes associated with vitellogenesis were revealed by the following paired t-tests (SAM) comparisons: a) two-month old vitellogenic (Vit2) females were compared with non-vitellogenic (NV) females, showing 825 differentially expressed transcripts during early stages of vitellogenesis, b) four-month old vitellogenic (Vit4) females were compared with NV females, showing 1,046 differentially expressed transcripts during vitellogenesis and c) E2-treated males were compared with control males, showing 1,828 differentially expressed transcripts regulated by E2. A Venn diagram revealed 822 common transcripts in the three groups, indicating that these transcripts were involved in vitellogenesis and putatively regulated by E2. In addition, 431 transcripts were differentially expressed in Vit2 and Vit4 females but not in E2-treated males, indicating that they were putatively not up-regulated by E2. Correspondence analysis showed high similarity in expression profiles of Vit2 with Vit4 and of NV females with control males. The E2-treated males differed from the other groups. The repertoire of genes putatively regulated by E2 in vitellogenic females included genes associated with protein synthesis and reproduction. Genes associated with the immune system processes and biological adhesion, were among the genes that were putatively not regulated by E2. E2-treated males expressed a large array of transcripts that were not associated with vitellogenesis.

The study revealed several genes that were not reported before as being regulated by E2. Also, the hepatic expression of several genes was reported here for the first time.

**Conclusion:**

Gene expression profiling of liver samples revealed 1,046 differentially expressed transcripts during vitellogenesis of which at least ~64% were regulated by E2. The results raise the question on the regulation pattern and temporal pleiotropic expression of hepatic genes in vitellogenic females.

## Background

The accumulation of yolk in oocytes of oviparous animals during oocyte development is essential for proper embryonic development after fertilization and is therefore, a key process in successful reproduction. In fish, the egg yolk protein precursors (vitellogenins) are synthesized in the liver, secreted to the plasma and transported to the oocytes for uptake in a process known as vitellogenesis. Several metabolic changes occur during vitellogenesis in the maturing female fish as reflected in the pronounced increase in liver weight, RNA content, lipid deposition, glycogen depletion, plasma proteins, calcium and magnesium and phosphoprotein content [1,3].

The most dominant trigger of *vitellogenin *(*vtg*) expression is the ovarian steroid hormone 17β-estradiol (E2) that is synthesized under the regulation of the hypothalamic–pituitary–gonad axis [reviewed in [4]]. Most data to-date supports the premise that the action of estrogens is mediated principally through specific nuclear Estrogen receptors (ERs). In the "classical" or "genomic" mechanism of E2 action, estrogens diffuse into the cell and bind to ERs, which are located in the cytosol or the nucleus of target cells. After ligand binding, the ERs form homo- or hetero dimers that bind to specific palindromic estrogen response elements (ERE) sequences [5] in the promoter region of estrogen-responsive genes, resulting in recruitment of coactivators or corepressors to the promoter. Subsequently this leads to increased or decreased mRNA levels and associated protein synthesis, resulting in the physiological response [6]. Two main ERs (ERa and ERb) were characterized in mammals, birds and fish. Three ER subtypes were described so far for fish and include the Estrogen receptor 1, Estrogen receptor 2b and Estrogen receptor 2a [with the gene names of *estrogen receptor 1 *(*esr1*), *estrogen receptor 2b (esr2b) *and *estrogen receptor 2a (esr2a)*, respectively) [7,9]. Some of the effects of estrogens are so rapid that they cannot depend on RNA and protein synthesis and are known as non-genomic actions. They involve activating protein-kinase cascades, leading eventually to regulation of gene expression through phosphorylation and activation of transcription factors (TFs) within the nucleus [10,12].

Hepatic expression of *vtg *is tightly coupled to E2-dependent up-regulation of *esr1 *expression [13,15]. Vtg is specific to maturing females and therefore assessment of *vtg *expression or Vtg plasma levels is considered a useful approach in evaluating female maturity related with peripheral gonadal steroid changes [16]. This protein is normally not detected in males or juveniles, but yolk precursor proteins can be detected in males or juveniles exposed to estrogens. Hepatocytes synthesize yolk precursor proteins when stimulated with exogenous estrogens or substances that mimic estrogens. Several changes in hepatic morphology such as proliferation of the endoplasmic reticulum and the Golgi apparatus also accompany estrogen stimulation. These aspects were investigated in several oviparous species [4,17-23].

Vtg is a large (MW; 250–600 kDa) and complex calcium-binding phospholipoglycoprotein and in order to reach the end product found in the plasma, substantial post-translational modification must occur within liver cells. First, the protein backbone of Vtg is synthesized on membrane-bound ribosomes and subsequently the Vtg molecule is lipidated, glycosylated and phosphorylated [Reviewed in [3]]. In addition, Vtg may carry additional compounds such as retinal that are also transported to the developing oocytes [24]. The genes involved in these processes have not been fully elucidated. In zebrafish, seven *vtg *genes were previously identified [25] but recent proteome profiling data from maturing ovarian follicles indicates the occurrence of eight *vtg'*s [26]. The proteins fall into three main families represented by Vitellogenin 1 or VtgAo1 (with five corresponding genes, *vtg 1, 4, 5, 6 *and *7*), Vitellogenin 2 or VtgAo2 (with two *vtg2 *genes) and Vitellogenin 3 or Vtg C (encoded by *vtg3*). Many more genes appear in the genome of zebrafish and fourteen of these genes were tightly linked to chromosome 22, while the phosvitinless gene (*vtg3*) was located on chromosome 11 [27].

In recent years, the study of hepatic expressed genes involved in fish vitellogenesis focused on few genes such as *vtg, esr1, insulin-like growth factor 1 *(*igf1*), *zona pellucida glycoproteins *(*zp's*), *choriogenin H, cytochrome p450, family 1, subfamily a *(*cyp1a*; also known as *cyp1a1*) and *peroxisome proliferator-activated receptors (ppar's) *[3,15,28-32] that are known to be regulated by estrogen. It is also well known that teleost apolipoproteins such as Apolipoprotein A-I (Apoa1), Apolipoprotein A-II (Apoa2) and Apolipoprotein E (Apoe) are regulated by E2 and presumably contribute to changes in the lipoprotein classes during vitellogenesis in fish [33,35]. A high-throughput expression genomics approach would provide complementary information to the single-gene approaches used so far. Large-scale microarrays, available for model fish species including zebrafish, provide the opportunity to simultaneously monitor the expression of thousands of genes in different physiological stages during vitellogenesis. This approach has already been used with success to elucidate the zebrafish embryonic transcriptome [36,37], to understand the molecular pathways defining gender specificity in zebrafish [38,39] and to explore hepatic gene expression after exposure to different estrogens [40,44].

The present study aims to identify genes involved in vitellogenesis and putatively regulated by E2 in the liver of zebrafish as a model fish, by using zebrafish oligonucleotide microarrays. Comparison of the hepatic expression profiles of vitellogenic and non-vitellogenic females provides information on the genes associated with vitellogenesis. In order to reveal E2-regulated genes, E2 treatment was administered to males for 48 hr at levels detected in the plasma of vitellogenic female. Genes suggested to be regulated by E2 were revealed by comparing the gene expression profiles of E2-treated males with those of vitellogenic females. The results also provide comparative information on the hepatic transcriptome profiles of 2- and 4- month old vitellogenic and non-vitellogenic females, E2-treated and control males and of the resemblance in gene expression profiles of these five groups. Distinctive putative pathways for the liver of vitellogenic females were found by analyses of the molecular functions and biological processes of the different treatment groups.

## Results

### Oocyte developmental stages in ovaries of vitellogenic and non-vitellogenic females

**Figure 1:**
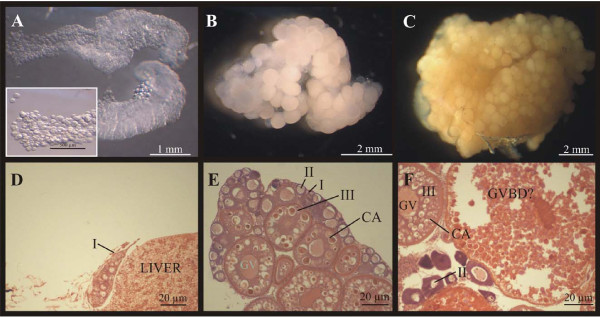
**Ovaries from zebrafish (*Danio rerio*)**. The top panels show ovaries from non-vitellogenic females (A), 2-month old (B) and 4-month old (C) females and the corresponding histological sections are shown in lower panels (D, E and F, respectively). **A) **A non-vitellogenic ovary containing transparent Stage I oocytes, shown at a higher magnification in the insert. **B) **A vitellogenic ovary collected from a 2-month old female. **C) **A vitellogenic ovary collected from a 4-month old female. **D) **Histological section of a non-vitellogenic ovary (I) and liver. **E) **A cross-section of an ovary from a 2-month old female showing oocytes at Stages I, II and III with cortical alveoli (CA). **F) **A cross-section of an ovary from a 4-month old female showing oocytes at Stages II and III and one oocyte possibly displaying germinal vesicle breakdown (GVBD). Cortical alveoli (CA) and the germinal vesicle (GV) are clearly shown in stage III oocytes.

Microscopic examination of the ovaries collected from non-vitellogenic (NV) females showed undeveloped, small sized ovaries containing transparent stage I oocytes (Fig. 1A, D). In comparison, ovaries collected from 2-month old (Vit2) fish contained oocytes at the perinucleolar stage (stage II) and oocytes with cortical alveoli, yolk bodies (stage III) and a conspicuous germinal vesicle (Fig. 1B, E). Ovaries from 4-month old females (Vit4) contained stage III but also larger stage IV oocytes and oocytes undergoing germinal vesicle breakdown (GVBD; Fig. 1C, F), carrying yolk bodies with crystalline yolk accrue.

### The association of E2 plasma levels with hepatic transcript levels of *esr1 *and *vtg3 *in the experimental groups

**Figure 2:**
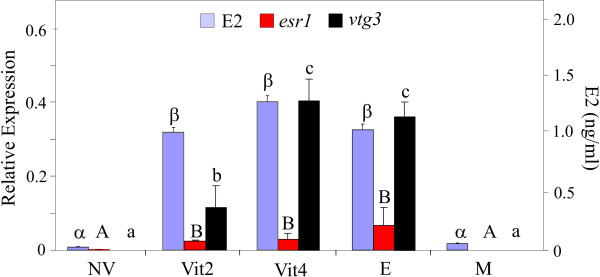
**Plasma levels of E2 and expression ratios of *esr1 *and *vtg3 *tested by RT-PCR**. Gene expression levels were normalized to *ef1a *and expressed as Mean +SD (n = 3 pools). The letters α and β indicate significant differences in the E2 levels. The letters A, B and a, b, c denote significantly different expression levels of *esr1 *and *vtg3*, respectively. E- E2-treated males, Vit2 or Vit4- 2- or 4-month old vitellogenic females, respectively, NV- Non-vitellogenic females, M- control males.

E2 levels increased in females according to their vitellogenic stage (Fig 2). The levels of E2 in plasma of vitellogenic females (Vit2 and Vit4) were similar to those of E2-treated males (p > 0.05). The E2 plasma levels of NV and of control males were significantly lower than those of vitellogenic females (Vit2 and Vit4) or E2-treated males (p < 0.01) and the plasma E2 levels of control males did not significantly differ from those of NV females (p > 0.05). Similar expression levels of *esr1 *were found by real-time PCR (Fig 2) in liver samples from Vit2 females, Vit4 females and E2-treated males (P > 0.05). The expression level of *esr1 *in these samples was significantly higher than those found in liver samples from NV females or control males (p < 0.05). The expression of *esr1 *was very low in the NV females and undetectable in the group of control males. Transcripts of *vtg3 *were detected only in vitellogenic females (Vit2 and Vit4) and E2-treated males. Expression levels of *vtg3 *were three fold higher in Vit4 females and E2-treated males than in Vit2 females (p < 0.01).

### Transcriptome analysis revealing gene expression patterns during vitellogenesis

Only 16,252 transcripts out of 16,399 were left for further analyses after processing the intensity values of each spot on the arrays and after normalization. SAM analysis revealed 2,523 transcripts (see Additional file 1) differentially expressed in livers of vitellogenic females (Vit4 and Vit2), NV females, E2-treated males and control males. The top 20 most differentially expressed transcripts (Table 1) included transcripts of *vtg3 *and *esr1 *that were used as markers for vitellogenesis and E2 treatment, respectively. The list also included transcripts of *vtg1, nothepsin *(*nots*), ESTs similar to *spectrin, nuclear envelope 1 *(*syne1*; AW019847), EST similar to f*ollistatin-like 1 *(*fstl1*; AI884233), EST similar to *nitric oxide synthase interacting protein *(*nosip*; AI545274) and EST similar to *glutamate receptor, ionotropic, kainate 1 *(*grik1*; BM071695), that were reported previously to be regulated by E2. The transcript of *vtg3 *(Table 1; see Additional file 1) was the most up-regulated transcript in the livers of E2-treated males and Vit2 or Vit4 females when compared with control males or NV females, respectively. The abundance of *vtg1 *transcripts was much lower, but significantly higher in E2-treated males than in vitellogenic females. A similar higher expression pattern of transcripts in E2-treated males vs. vitellogenic females (Table 1) was found for *nots, fstl1 *and EST similar to *lectin mannose- binding 1 precursor *(*lman1*; AW175541*)*. Several transcripts were more abundant in vitellogenic females (Vit2 or Vit4) than in E2- treated males, including the gene *ankyrin *(*ank*), transcripts of EST similar to *ectonucleoside triphosphate diphosphohydrolase 4 *(*entpd4*; AW344063) and an EST similar to f*amily with sequence similarity 20C *(*fam20c*) (Table 1).

**Table 1:** A list of 20 most regulated annotated genes during vitellogenesis and after E2 treatment of males.

	Description	Symbol	GeneBank	Vit4/NV	Vit2/NV	E/M	Function according to gene
1	Vitellogenin 3, phosvitinless	vtg3	AF254638	103.3	94.5	125.7	Egg yolk precursor, phosvitinless.
2	Similar to reticulon 1	rtn1	BI983061	75.5	88.5	79.3	Associates with the endoplasmic reticulum.
3	Nothepsin, cathepsin e	nots	AJ278269	76.6	76.8	86.8	Eukaryotic aspartyl (acid) protease.
4	Similar to low density lipoprotein receptor	ldlr	BF717943	28.3	28.2	136.3	Plays a central role in cholesterol metabolism.
5	Moderately similar to spectrin repeat containing, nuclear envelope 1	syne1	AW019847	16.1	21.2	14.7	Involved in cytoskeletal structure.
6	Similar to ectonucleoside triphosphate diphosphohydrolase 4	entpd4	AW344063	97	70.2	25.5	Cleaving nucleotide tri- and diphosphates.
7	Similar to follistatin-like 1	fstl1	AI884233	NS	NS	3.2	Play an important role in tissue specific regulation.
8	Similar to human Prefoldin subunit 6	pfdn6	BI891290	1.5	NS	3.7	Binds and stabilizes newly synthesized polypeptides.
9	Lag1 homolog, ceramide synthase 2	lass2	AW171063	1.8	NS	4.5	May play a role in the regulation of cell growth.
10	Ankyrin repeat domain 6	ank	AF395113	12.3	10.3	4.3	Ankyrin repeats mediate protein-protein interactions.
11	Similar to nitric oxide synthase interacting protein	nosip	AI545274	8.1	7.4	11.6	Promotes translocation of eNOS from the plasma membrane to intracellular sites.
12	Similar to heterogeneous nuclear ribonucleoprotein h1	hnrnph1	AI497414	1.7	NS	2.8	RNA recognition/binding motif.
13	Similar to family with sequence similarity 46 c	fam46c	AI959558	4.5	4.7	14	The function of this gene is unknown.
14	Vitellogenin 1	vtg1	AF406784	7	6.3	28.6	Egg yolk precursor.
15	Similar to human guanylate binding protein1	gbp1	BM181499	8.5	8.2	2.7	Specifically bind guanine nucleotides (GMP, GDP, and GTP).
16	Similar to human protein fam20c precursor?	fam20c	BF717944	33.2	35.2	20.8	Has a crucial role in normal bone development.
17	Similar to glutamate receptor, ionotropic, kainate 1	grik1	BM071695	NS	NS	-16.4	Ligand-gated ion channel.
18	Similar to lectin, mannose-binding, 1 precursor	lman1	AW175541	4.8	4	6.6	Mannose-specific lectin, a member of a Mannose-specific lectin, a member of a the secretory pathway of animal cells.
19	Similar to rna polymerase ii associated protein 1	rpap1	BM157210	NS	NS	2.4	The function of this gene is unknown.
20	Estrogen receptor 1	esr1	AF349412	16	15.5	17	A ligand-activated transcription factor.

Regulation during vitellogenesis is indicated as the ratio between Vit4/NV and Vit2/NV.

Regulation by E2 is indicated by the ratio E/M. A weak similarity is indicated by a question mark (?). For the complete list of the regulated genes see Additional file 1.

**Figure 3:**
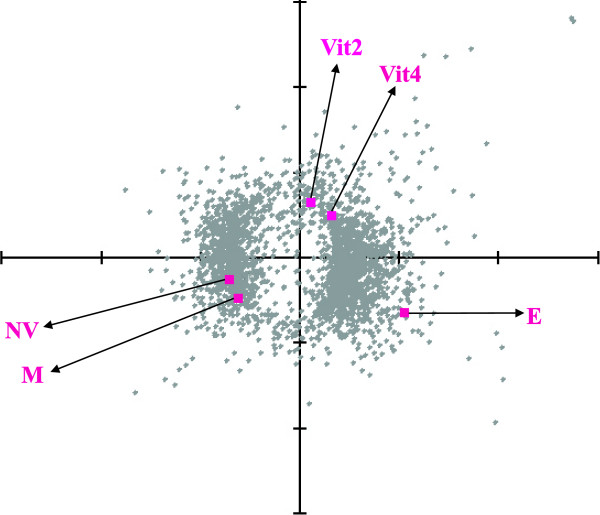
**Correspondence analysis showing the association in the expression patterns of the five studied groups**. Correspondence analysis (COA) for the 2, 523 differentially expressed transcripts found by SAM analysis presenting the association between the expression patterns and the five tested groups: Vit2 and Vit4- 2- or 4-month old vitellogenic females, NV- non-vitellogenic females, E- E2-treated males, M- control males. Only the middle part of the resulted graph is shown (four genes are missing).

Correspondence analysis (COA) was performed to study the associations between the 2,523 differentially expressed transcripts in the five tested groups (Fig 3). The results indicated close association and sharing of the same expression pattern of Vit2 with Vit4, and of NV females with control males. The group of E2-treated males did not show any association with the other groups. Most of the transcripts in the groups of E2-treated males and vitellogenic females (Vit2 and Vit4) were up-regulated in comparison with NV and control males (Fig 3).

**Figure 4:**
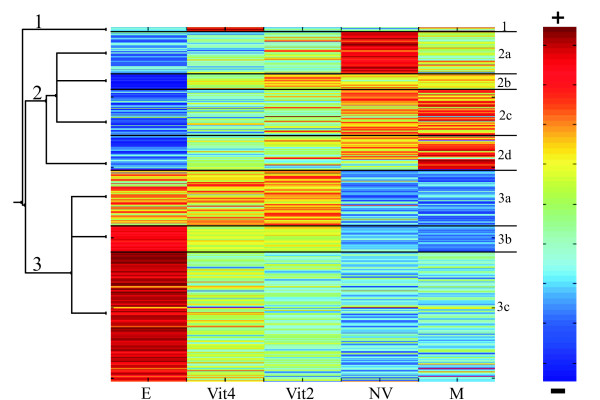
**Coupled Two Way Clustering Analysis of the five tested groups**. Coupled Two Way Clustering representing 2, 523 transcipts with significantly altered expression, found by SAM analysis (0.1% FDR) for all five tested groups: Vit4 and Vit 2- 4-or 2-month old vitellogenic females, NV- non-vitellogenic females, E- E2-treated males, M- control males. Genes and groups are clustered for similar expression profiles and colors show the expression levels in each specific group, with red representing the highest expression level and blue the lowest expression level (according to the right hand scale). Three main clusters are marked by numbers 1 to 3 and their sub-clusters are separated by black lines and indicated by letters.

Coupled Two-Way Clustering of the 2,523 differentially expressed transcripts was performed to reveal similarity in the expression patterns in the five tested groups. Three main clusters were revealed (Fig 4): 1) A small cluster of 34 transcripts with high expression in Vit4 females and low expression in other samples; 2) A cluster of 985 transcripts (Fig 4; subdivided into 2a, 2b, 2c and 2d) showing high expression patterns in control males and NV females, lower expression in the two groups of vitellogenic females (Vit2 and Vit4) and the lowest expression level in E2-treated males; and, 3) A cluster of 1,504 transcripts (Fig 4; subdivided into 3a, 3b and 3c) with high expression levels in E2-treated males, low expression levels in control males and NV females and interim expression levels in Vit2 and Vit4 females. Closer examination revealed that in some sub-clusters the expression pattern of Vit2 females was similar to that of NV females, while in others it was similar to Vit4 females. The expression patterns of Vit2 females were at an intermediate stage between those of the older vitellogenic females (Vit4 females) and NV females. This pattern corresponds with the expression levels of *vtg3 *in these groups mentioned previously (Fig 2).

#### Genes suggested to be regulated by E2 during vitellogenesis

**Figure 5:**
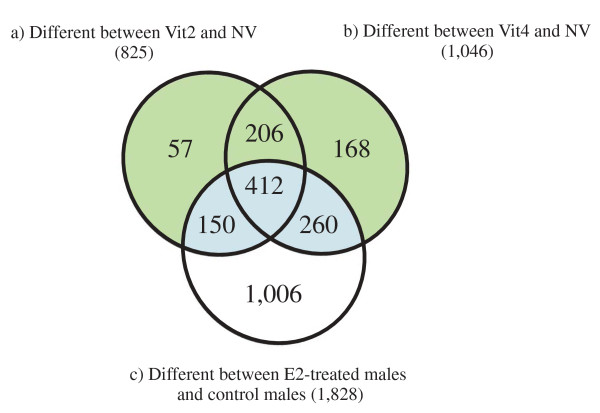
**Number of transcripts putatively associated with vitellogenesis or E2 treatment of males**. A Venn diagram showing the number of transcripts differing significantly between vitellogenic and non-vitellogenic females and putatively associated with E2 regulation: **a) **Transcripts of genes significantly different between two-month old vitellogenic females (Vit2) and non-vitellogenic females (NV). **b) **Transcripts of genes significantly different between four-month old vitellogenic females (Vit4) and NV, and **c) **Transcripts of genes significantly different between E2-treated males and control males. Combination of these three comparisons by Venn diagram illustrates the number of genes differing during vitellogenesis and putatively regulated by E2 (colored pale blue), genes differ during vitellogenesis putatively not directly regulated by E2 (colored light green) and genes unique to males (colored white).

Additional analyses of the 2,523 transcripts mentioned above, were performed to identify genes with significantly different expression levels between vitellogenic and non-vitellogenic females and regulated by E2. The following paired t-tests (SAM) comparisons were performed (Fig 5): a) Vit2 females were compared with NV females showing 825 differentially expressed transcripts; b) Vit4 females were compared with NV females showing 1,046 differentially expressed transcripts; and, c) E2-treated males were compared with control males showing 1,828 differentially expressed transcripts. A Venn diagram revealed 822 common transcripts in the three groups that were suggested to be regulated by E2 (Fig 5; colored blue), 431 transcripts that were putatively not regulated by E2 (Fig 5; colored green) and 1,006 transcripts that were unique for males (Fig 5; colored white).

**Figure 6:**
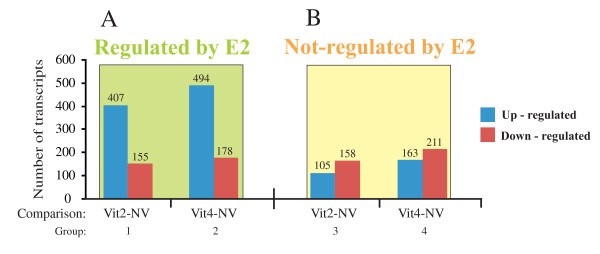
**The number of up- or down-regulated trancripts that were putatively regulated by E2 during vitellogenesis**. The number of transcripts of vitellogenic females (Vit2 or Vit4) differing from non- vitellogenic females (NV). A) Number of transcripts putatively regulated by E2. B) Number of transcripts putatively not regulated by E2. The lists of genes for each comparison are shown in detail in Additional file 2, according to the group numbers.Vit2 and Vit4 females are 2- and 4- month old females, respectively. The number of up-regulated or down regulated transcripts for each group is shown on the top of the columns.

In order to reveal genes associated with vitellogenesis, vitellogenic females (Vit2 or Vit4) were compared with NV females by paired t-test analysis (SAM) (Fig 6; see Additional file 2). In general, the number of E2 regulated transcripts was higher in Vit4 (672 transcripts) than Vit2 (562 transcripts), when compared with NV females. Relatively more transcripts were E2 up-regulated than down-regulated (Fig 6A).

#### Genes putatively not directly regulated by E2 during vitellogenesis

Paired t-test analysis (SAM) comparing Vit2 or Vit4 females with NV females revealed 263 or 374, respectively transcripts that were not regulated by E2 (Fig 6B; see Additional file 2). The proportion of the down-regulated transcripts was higher (60.0% and 56.4% for Vit2 and Vit4 females, respectively) than the up-regulated transcripts indicating a slightly reverse trend from the E2 regulated genes.

#### Comparison between E2-treated males, control males and females

**Figure 7:**
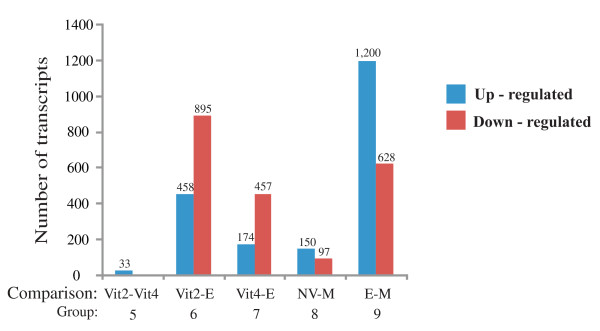
**The number of up- or down-regulated transcripts revealed by paired t-test (SAM) comparisons of the studied groups**. Paired t-test comparisons, showing the number of transcripts differing significantly between: i) two vitellogenic female groups (Vit2 and Vit4), ii) between E2-treated males (E) and vitellogenic females (Vit2 and Vit4), iii) between control males (M) and non-vitellogenic females (NV), and iv) between E2-treated males and control males. Vit2 and Vit4 females are 2- and 4-months old females, respectively. The lists of genes for each comparison are shown in detail in Additional file 2, according to the group numbers. The number of up-regulated or down regulated transcripts for each group is shown on the top of the columns.

E2-treated males were compared with control males, showing 1,828 differentially expressed transcripts (Fig 5c) and most of them were up-regulated (Fig 7; see Additional file 2). A smaller number of expressed transcripts differentiate E2-treated males from Vit4 than Vit2 females, indicating a higher similarity between E2-treated males and Vit4 females. Most of these transcripts were down regulated in vitellogenic females. Moreover, higher resemblance of NV females to control males was revealed by the relatively small number of differentially expressed transcripts in NV (Fig 7; see Additional file 2). Only 33 transcripts differ in their expression levels between Vit4 and Vit2 females.

### Putative processes associated with vitellogenesis using Gene Ontology terms

**Figure 8:**
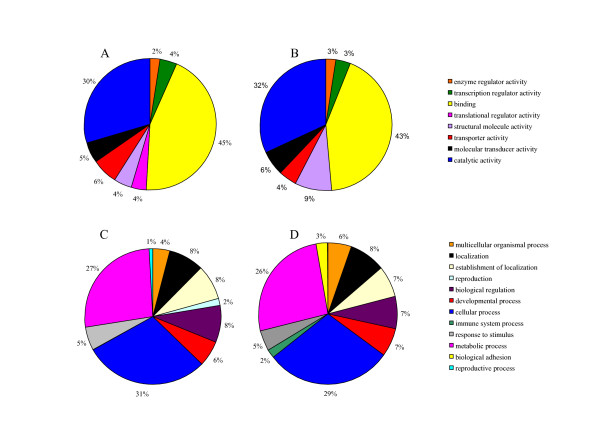
**Molecular functions and biological processes of genes putatively regulated by E2 or not regulated by E2**. Ontology pies created by the Blast2GO program, according to the genes ontology (GO) of the annotated genes (level 2 terms). Putative molecular functions are shown in pies A and B, and biological processes are shown in pies C and D. The pies represents comparison between transcripts regulated by E2 (pies A and C) or those not regulated by E2 (pies B and D).

Analyses of the molecular functions and biological processes were performed for all 2,523 transcripts corresponding to the differentially expressed transcripts. Only 607 genes were found as annotated among the 1,255 transcripts that were regulated in vitellogenic females (Vit2 and Vit4). In general, similar molecular functions and similar relative abundance (in percent) were found among the putatively E2 regulated genes and genes not regulated by E2 (Fig 8A, B). An exception includes genes associated with translational regulatory activity that were found only in the group putatively regulated by E2, indicating the prominent position of proteins synthesis during vitellogenesis. Analysis of the biological processes shows the occurrence of processes associated with reproduction only in the E2 regulated group and of immune system processes and biological adhesion only in the group of genes not regulated by E2 (Fig 8C, D). Other biological processes were similar among the putatively E2 regulated genes and those not regulated by E2. Some more detailed information on the number of transcripts and their descriptors that were associated with some selected functions, are shown in Table 2 and in Additional file 3, respectively.

**Table 2:** The number of genes regulated or not-regulated by E2 in selected GO functions.

**GO No**	**Description**	**E2-regulated**	**Not regulated by E2**
GO:0042562	Hormone binding	3	2
GO:0015485	Cholesterol binding	2	1
GO:0006629	Lipid metabolic process	15	8
GO:0008289	Lipid binding	8	1
GO:0006955	Immune response	4	3
GO:0002376	Immune system process	0	7
GO:0007155	Cell adhesion	0	10
GO:0003700	Transcription factor activity	22	7

Number of genes putatively regulated or not-regulated by E2, found in selected GO functions. The list of genes matching each function is shown in Additional file 3.

### Validation of microarray results

Validation of the microarray results was performed by testing the relative expression of 16 genes (see Additional file 4) in the same RNA samples that were used for the chip hybridization, by real-time PCR. The 16 tested transcripts were: 1) genes highly expressed in vitellogenic females and known to be induced by E2 treatment [*esr1, vtg1, vtg3*, *nots *and *cytochrome p450 2k6 *(*cyp2k6*)], 2) genes that were highly expressed in males and known to be down-regulated by E2 (*cyp1a1 *and *igf1*), and 3) genes that showed significantly different expression levels in the tested groups [*retinoic acid receptor alpha a *(*raraa*)*, alcohol dehydrogenase 5 (adh5), alcohol dehydrogenase 8b *(*adh8b*)*, igf1 and retinol dehydrogenase *10 (*rdh10*), *retinol dehydrogenase *14 (*rdh14*), *dehydrogenase/reductase (SDR family) member 10 *(*dhrs10*), *stearoyl-desaturase (sCd)*, *fatty acid desaturase 2 *(*fads2*) and *steroidogenic acute regulatory protein *(*star)*]. A very high correlation was found between the microarray and real-time PCR results, with regression coefficients (R^2 ^values) ranging from 0.9102 to 0.9340 (see Additional file 5).

## Discussion

This study aims at identifying and characterizing genes associated with vitellogenesis and defining the role of E2 in their regulation by using five physiological groups: non-vitellogenic females, vitellogenic females (2- and 4-month old), E2-treated males and control males. The expression levels of *esr1 *and *vtg3 *corresponding with the E2 plasma levels of females and E2 treated males, confirmed the efficacy of the E2 treatment. They also depicted the physiological state of the vitellogenic females, indicating that Vit2 females were at an interim stage between NV and Vit4 females.

The results provided a novel insight into the number and scope of hepatically regulated genes during vitellogenesis and indicated that most genes were regulated during the early stages of the process as young vitellogenic females (Vit2) differed by only 33 transcripts from older females (Vit4). Moreover, only ~64% of the transcripts regulated during vitellogenesis were suggested to be also regulated by E2. The resemblance in the gene expression pattern between non-vitellogenic females and males, stresses the specific change in pattern taking place during vitellogenesis in females. This change cannot be simply attributed to E2 as E2 treatment of males at physiological concentrations, resulted in a 1.8 fold higher number of genes than those regulated during vitellogenesis. These results also emphasize that the wide effects of xenobiotics with estrogen activity [45,47] are not confined to genes associated with oocyte development. The following discussion sections highlight selected specific genes and putative pathways that were regulated during vitellogenesis and E2 treatment of males. Some of these putative pathways, were previously shown to be regulated in E2- treated males [41].

### A list of 20 most differentially expressed hepatic transcripts reveals genes regulated by E2 and novel hepatic transcripts

The list of the 20 most differentially expressed hepatic transcripts includes genes known to be regulated by E2, genes that were not recorded previously as regulated by E2 or genes that were not previously reported to be expressed in the liver. Eight of the 20 most differentially expressed genes (Table 1), were previously reported to be regulated by E2, including: *vtg1 and vtg3 *[25,48-50], *nots *[42,51], *syne1 *[52], *fst1 *[53], *nosip *[54], *grik1 *[55] and *esr1 *[15,49]. Transcripts that were not associated previously with E2 regulation include *reticulon1 *(*rtn1*), *entpd4 *and *lman1. *A few transcripts (*syne1*, *fstl1 *and the *fam20c*) were not reported previously in hepatic cells. Proteins associated with cytoskeleton formation (*syne1 *and *ank) *showed higher up-regulation in vitellogenic females than after E2 treatment of males, supporting a putative role in the growth of the liver [56] and of the dramatic increase in protein synthesis and secretion by the endoplasmic reticulum [3] during vitellogenesis.

Zebrafish display eight different variant gene sequences [25,27] for *vtg *genes but the array used here included probes only for *vtg3 *and *vtg1*. Since *vtg1 *shows high sequence similarity with *vtg4*, *vtg5*, *vtg6 *and *vtg7 *(all coding for VtgAo1), transcript levels for *vtg1 *may also reflect the expression of these genes. The higher expression levels of *vtg3 *compared with *vtg1*, may suggest their differential regulation by E2 stems from differences in the estrogen response elements (ERE's) in the promoter regions [42].

### Putative molecular functions and biological processes regulated during vitellogenesis and by E2 treatment

Numerous prominent and putatively regulated functions were revealed in this study to take place during vitellogenesis and include lipid metabolism and lipid binding, hormone and cholesterol binding, transcription factor activity, immune response, immune system processes and cell adhesion (Table 2 and see Additional file 3). The task of allocating processes and functional significance to genes that were putatively regulated by E2 and also to those that were putatively not regulated by E2, was faced with a general difficulty for zebrafish. Linking specific pathways or modes of function with genes by the general descriptors provided by Gene Ontology (GO) for zebrafish is problematic due to the incomplete annotation of the zebrafish genome and a deficiency in functional studies. Consequently, several GO terms rely on homology of putative functions described in higher vertebrate species.

#### Lipid metabolism associated with vitellogenesis and E2-treatment of males

Several genes with a role in lipid metabolism were reported to be regulated by E2 [57,58]. Genes associated with lipid metabolic processes putatively regulated in vitellogenic females or putatively regulated by E2, were also identified in the present study. Transcripts indicating a change in plasma lipoproteins were identified here, supporting previous published results on higher levels of plasma lipoproteins during vitellogenesis in fish [34,35]. Plasma lipoproteins associated with transport of lipids are mainly synthesized in the liver and intestine [59]. The protein components of lipoproteins, the apolipoproteins, form distinct complexes and two gene clusters, one consisting of *apoa1*, *apoa4 *and *apoc3 *and the other of *apoe *(*apoeb *for zebrafish), *apoc*, *apoc2 *and *apoa4*. These apolipoproteins are known from mammals and some were also characterized in fish [60]. In the current study, there were no significant changes in *apoa1 *gene expression, in contrast with previous studies reporting on the downregulation of *apoa1 *gene expression after E2 and EE2 treatment [34,42,43]. Interestingly, Apoa1 was also found to serve as an antimicrobial protein and to be associated with the immune response system in fish [61]. However, the expression of *apoa4 *and *apoeb *was down-regulated in vitellogenic females in comparison to NV females or after E2 treatment of males (see Additional file 1 and Additional file 3).

Several genes in the lipid metabolic processes are associated with the PPARs signaling pathway [62,63] and some of them were found to be regulated in this study. The Ppars are ligand-inducible TFs belonging to the nuclear hormone receptor superfamily and are important regulators of lipid and energy homeostasis. Three isotypes have been identified in mammals, birds and amphibians, termed Ppar alpha (Ppara), Ppar beta (Pparb) (also known as Ppar delta) and Ppar gamma (Pparg) and each isotype is a product of one gene and shows distinct tissue distribution [63]. Ppara functions in regulating reversible induction of β-oxidation in specific tissues but mainly the liver [62]. Unexpectedly, there was no significant change in the expression of *ppara *in the liver of vitellogenic females or E2-treated males. The Pparb presumably functions in global control of lipid homeostasis and cellular proliferation and differentiation in mammals, is expressed in the liver and is moderately activated by a range of unsaturated fatty acids. Multiple/isoforms genes were found for *pparb *in several fish species, with two genes for *pparb *in zebrafish [64,67]. The *pparb1 *was up-regulated in Vit4 females and control males while *pparb2 *was down-regulated in Vit4 and Vit2 females and by E2 treatment. In mammals, Pparg is associated with fat accumulation, particularly in adipocytes and in lipid accumulation in macrophages [62]. Here, the *pparg coactivator related 1 *(*ppargcr1*) was up-regulated in Vit4 females and after E2 treatment (see Additional file 1).

A large array of transcripts putatively associated with lipid metabolism, was found to be regulated during vitellogenesis and by E2 treatment (see Additional file 3). Among them were genes coding for enzymes associated with fatty acid elongation and metabolism, and bile acid metabolism. Also, several transcripts of putative members of the cytochrome 450 superfamily were found to be regulated during vitellogenesis. This group of monooxygenases catalyzes several reactions involved in xenobiotic and drugs metabolism, synthesis of cholesterol, steroids and lipids.

#### Biosynthesis and catabolism of steroids

**Figure 9:**
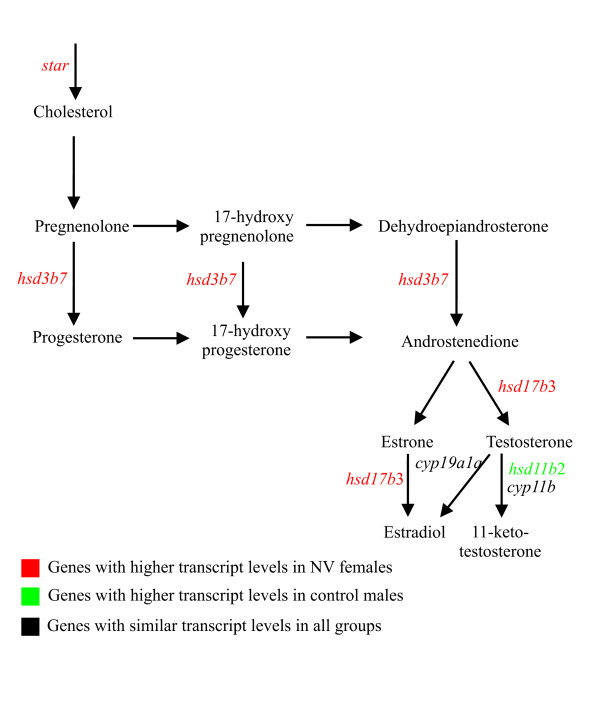
**The steroid synthesis pathway showing genes regulated in this study**. The pathways of steroid synthesis showing the expression patterns of transcripts associated with the different biosynthesis steps. Through a series of enzyme reactions, cholesterol is converted into testosterone (T), which is subsequently converted either to E2 or to 11-ketotestosterone (11-KT). For a detailed explanation see the text. *hsd3b7 - hydroxyl-delta- 5 steroid 3 beta *and *steroid delta-isomerase7, hsd17b3 - hydroxysteroid 17b dehydrogenase, hsd11b2 - hydroxysteroid 11b dehydrogenase 2*, *star - steroidogenic acute regulatory protein*, *cyp19a1a - cytochrome p450 19a*, and *cyp11b - 11 beta hydroxylase*.

Numerous genes involved in biosynthesis and inactivation of steroids were regulated during vitellogenesis (Table 2, see Additional file 1 and Additional file 3), suggesting that biosynthesis and catabolism of steroids takes place in the liver. Endocrine regulation of this pathway in the zebrafish liver was reported previously after EE2 treatment [43]. Transcripts previously identified with a role in E2 and 11-KT synthesis, were found to be regulated during vitellogenesis and by E2, as shown in Fig 9. In this pathway, the first and rate limiting step in steroid synthesis is the transport of cholesterol to the inner mitochondrial membrane by *star *[68,70]. Subsequently, through a series of enzyme reactions (Fig. 9), cholesterol is converted into testosterone (T) and forms either E2 or 11-KT, as it occurs in fish gonads [71]. The hepatic expression of *star *was previously reported only for human liver [72] and E2 was involved in down regulating its expression in other tissues [73,74]. Here we show the expression of *star *in the zebrafish liver (see Additional file 5) and surprisingly it was highly expressed in NV females and putatively not regulated by E2 (see Additional file 3). The expression patterns of two additional genes coding for enzymes involved in steroid metabolism were also significantly higher in NV females (Fig. 9). These include an EST similar to *hydroxyl-delta-5 steroid 3 beta *and *steroid delta-isomerase7 [(hsd3b7; *AW133873, AW116229), see Additional file 3] and an EST similar to *hydroxysteroid (17-beta) dehydrogenase 3 *[(*hsd17b3*; AW595044), see Additional file 1]. The enzyme Hsd3b7, catalyzing the conversion of pregnenolone to progesterone (Fig. 9) was found to accept in zebrafish several androgens as substrates, including androstenedione, epiandrosterone and dehydroepiandrosterone [75]. The synthesis of 11- KT from testosterone, a potent androgen in male teleosts [76] probably involved the upregulation of *hydroxysteroid 11-beta dehydrogenase 2 *(*hsd11b2*) in males (see Additional file 1).

One of the explanations for the higher transcript levels of *star*, *hsd3b7 *and *hsd17b*3 in NV females, could be related to the variable levels of steroid hormones in the plasma of females during onset of vitellogenesis [77,78].

#### Hormone binding and TFs

The process of vitellogenesis is known to be regulated by E2 through the induction of *esr1*, as mentioned before. A similar putative expression pattern for genes coding for two additional hormone binding receptors was suggested to take place during vitellogenesis and regulated by E2 (Table 2, see Additional file 3). These include the *progesterone receptor membrane component 2 *(*pgmrc*) and the *kappa opioid receptor 1 *(*oprk1*) as shown by the regulation of their respective ESTs; an EST similar to *pgmrc2 *(AW153364) and an EST similar to *oprk1 *(BG883146). Transcripts of *pgmrc *genes were found in livers of mammals and oviparous vertebrates including zebrafish but their functions remain unknown [79,80]. The gene *pgmrc2 *is known to be involved in progestin signaling in several vertebrate reproductive tissues and in the brain (reviewed in [79]) and Pgmrc1 (closely related to Pgmrc2) was suggested to have a role in the regulation of oocyte maturation in trout ovarian follicles [80]. Since PGMRC1 was suggested to be regulated by testosterone in porcine hepatocytes [81], the high transcript levels of *pgmrc2 *found in vitellogenic females could be linked with the elevated testosterone levels associated with higher E2 plasma levels [77,78]. The opioid receptors have multiple effects on reproductive, endocrine and immune functions. Transcripts of *oprk *were found to be widely expressed in rat tissues including in the liver, yet their function in the liver remained unknown [82].

**Figure 10:**
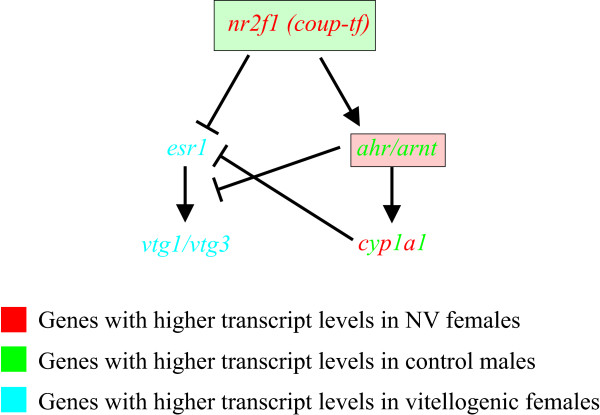
**A suggested cross-talk between Aryl hydrocarbon receptor (Ahr) and Er1 signaling pathways**. A cross talk is suggested by the expression of the following genes: *esr1, vtg1 *and *vtg3*, *nuclear receptor subfamily 2f1 *(*nr2f1*; similar to human COUP-TF)*, ahr-nuclear translocator2 (arnt2), cyp1a1 *and *ahr2*. The Nuclear receptor 2f1 (Nr2f1) inhibits E2-induced reporter gene and regulates transcription activated by the Ahr. Upon binding of the ligand, Ahr is translocated into the nucleus where it dimerized with the Ahr nuclear translocator (Arnt). The Ahr/Arnt complex binds with high affinity to specific xenobiotic response elements (XREs) and activates the transcription of enzymes such as Cyp1a1 (Cytochrome p450 1a1), involved in the metabolism of many drugs and xenobiotics. A negative correlation was found between the expression patterns of the genes *esr1 (estrogen receptor 1), vtg1 *and *vtg3 (vitellogenin1 and vitellogenin3, respectively) *and the genes *nr2f1, arnt2, cyp1a1 *and *ahr2. *The following expression patterns were found: i) *esr1, vtg1 *and *vtg3 *were highly expressed in vitellogenic females and E2-treated males; ii) *ahr2 *and *arnt2 *were highly expressed in control males and showed higher (though not significantly different) expression levels in non-vitellogenic females than vitellogenic females; iii) *cyp1a1 *was highly expressed in non-vitellogenic females and control males and significantly down-regulated by E2 treatment, and iv) *nr2f1 *was highly expressed in non-vitellogenic females and showed higher expression levels (though the difference was not significant) in control males than E2-treated males.

A cross-talk between the signaling pathways of two TFs, the Esr1 and the Aryl hydrocarbon receptor (Ahr), was suggested to occur during vitellogenesis based on the expression patterns of genes involved in these pathways, implying a possible regulation of Vtg synthesis (Fig. 10). AhR is a ligand activated TF involved in xenobiotic and drug metabolism in the liver [83]. A cross-talk between AhR and ER1 signaling [84] was shown to be regulated through the nuclear receptor COUP-TF by inhibiting E2-induced genes and regulating AhR activated transcription [85,86]. Also, ligand activated Ahr inhibited Esr1 from initiating *vtg *transcription and blocked the auto-regulatory loop of the *esr1 *gene expression, in primary cultures of hepatocytes [31,46,87]. Furthermore, the effectiveness of the different Ahr-ligands of inhibiting Vtg synthesis, was directly related to their ability of inducing higher Cyp1a protein levels and consequently its enzymatic activity [31,46,87]. So far, the effects of activation of piscine AhR on the hepatic expression of *esr1 *and *vtg *were studied after exposure to environmental pollutants and different estrogens [31,46,87-91]. Unexpectedly, in the current study, expression patterns for genes in this pathway were associated with gender and the occurrence of vitellogenesis in females and not specifically with E2 treatment. The crosstalk between the ER alpha and AHR signaling pathways (Fig 10) was suggested by the expression patterns found for the following genes: *esr1, vtg1 *and *vtg3, nuclear receptor subfamily 2f1 *(*nr2f1*; similar to human COUP-TF)*, ahr-nuclear translocator2 (arnt2) *(all in see Additional file 3)*, cyp1a1 *and *ahr2 *(see Additional file 1). Presumably, this crosstalk may be one of the mechanisms preventing Vtg synthesis in males and non-vitellogenic females. Accordingly, it could be speculated that an Ahr-ligand should be found in the plasma of males and non-vitellogenic females. If activated, the Ahr pathway will inhibit *esr1 *transcription and consequently Vtg synthesis, but with the onset of vitellogenesis in females, the effect of this pathway will be reduced and permit the synthesis of Vtg.

#### Immune system processes and immune response

Surprisingly, all transcripts related to immune system processes in this study, were down-regulated in vitellogenic females and putatively not regulated by E2 (Fig 8). Three of these transcripts are also related to the immune response GO term (Table 2, see Additional file 3). The other transcripts related to the immune response term were all up-regulated by E2 treatment and some were also up-regulated during vitellogenesis (Table 2, see Additional file 3). This is in contrast to previous studies where E2 treatment repressed the expression of immune system and immune response related transcripts [92,93]. Down-regulation in transcript levels in vitellogenic females (but not regulated by E2), was observed for genes coding for a class of intracellular molecules that play a role in coupling T-cell antigen receptor stimulation to the activation of integrins, *major histocompatibility complex *class genes (*mhc*'s), proteases mediating programmed cell death or apoptosis [*caspase 8 *(*casp8*)] and a gene coding for an adherens junction protein [*catenin beta 1 *(*ctnnb1*)], functioning in communication and adhesion between cells, and anchoring the actin cytoskeleton. The genes showing high transcript levels in vitellogenic females and E2 treated males include an interferon activated gene, a TF that regulates *mhc class II *genes and the *oprk1 *previously described to be affected by steroid hormones, including E2 [94,95].

### Conclusion

Microarray profiling of liver samples revealed expression patterns characteristic of vitellogenic females of which only ~64% were found to be putatively regulated by E2. The repertoire of regulated genes implicates a wide range of functions especially those associated with protein synthesis, lipid metabolism, steroid biosynthesis, hormone binding and TF. Genes associated with the immune system and biological adhesion were among the genes that were up-regulated in vitellogenic females but not in E2-treated males, indicating that they were putatively not regulated by E2. E2-treated males expressed a large array of genes that were not associated with vitellogenesis. The study revealed several genes (*rtn1, entpd4, lman1*) that were not reported before as being regulated by E2. Also, the hepatic expression of several genes (*e.g.*, *syne1, fstl1, fam20c, star*) is reported here for the first time for fish liver. In general, these results raise the question on the identity of the factors that regulate the pleiotropic expression of hepatic genes in vitellogenic females, in addition to E2.

## Methods

### Animals

Zebrafish were purchased from a local fish supplier (A&H Holdings, Israel LTD). All fish were maintained in 5-liter aquaria with UV treated, recycled and dechlorinated water and at ambient temperature of 25 ± 2°C with a light/dark cycle of 14/10 hr. The fish were fed twice a day, with shrimp nauplii (PGT, Eilat, Israel) in the morning and fish eggs in the afternoon. Non-vitellogenic females were kept under a light/dark cycle of 6/18 hr and fed twice a day with dry pellet food to avoid access to steroid compounds that maybe found in live food. For histological analysis the fish body or ovaries were fixed in Bouin's. Fixation, sectioning, and histological examination were performed according to [96]. Paraffin sections of 4–7 µm were stained with hematoxylin and eosin. The terminology used by [97] for the zebrafish was adopted for this study. All fish were anaesthetized with Tricaine (Sigma-Aldrich, USA) before experimental procedures [98] and treatment of fish adhered with institutional regulations.

### Samples collection and 17β-estradiol treatment

Two experiments were performed, one for evaluating the effect of E2 exposure in males and a second experiment for comparing gene expression in the liver of non-vitellogenic females with that of vitellogenic females. The experiments designs were as follows: 1) Four months old zebrafish were divided into three groups consisting of 8 fish in each group: i) adult spermeating males weighing 2.45 ± 0.207 g (N = 8) were exposed to E2 (Sigma-Aldrich, USA) by immersion for 48 hr (group E2-treated males). The concentration used was 5 μg/L (18 nM), as 3–4 ng/ml was determined to be the E2 natural concentration in the plasma of adult vitellogenic female ZF [99]. A period of 48 hr of exposure was chosen as the highest expression level of *esr1 *was reached after 12 hr [100] and the expression of *vtg *stabilized after 48 hr of exposure to E2 and lasted for 17 days [50]. The hepatosomatic index (HIS) of E2-treated males was 5.6 ± 0.6 after treatment. ii) control males weighing 2.26 ± 0.246 g (N = 8;) and a HIS of 3.2 ± 0.4; iii) vitellogenic females weighing 3.95 ± 0.303 g (N = 8) and showing a HIS of 5.9 ± 0.4 (group Vit4). Four replicate samples were prepared for each group and each replicate consisted of a pooled sample from livers of two fish. The samples were pooled after RNA extraction (see below). The fish in this experiment were kept with a light/dark cycle of 14/10 hr. The fish were fed twice a day, with shrimp nauplii (PGT, Eilat, Israel) in the morning and fish eggs in the afternoon. 2) In order to reveal the differences between vitellogenic and non-vitellogenic females, a second experiment was designed. One month old zebrafish were divided into two groups consisting of 32 fish in each group. Due to the small size of the liver, pooled samples from eight fish were prepared for each of the four replicates in the expression studies. The pooling of samples was done after total RNA extraction (see below). The groups consisted of: i) fish that were kept under a light/dark cycle of 14/10 hr for 5 weeks weighing 2.56 ± 0.426 g (N = 32) and a HIS of 5.6 ± 0.4, with ovaries in vitellogenic stage (group Vit2). Fish were kept at 25 ± 2°C and fed twice a day, with shrimp nauplii (PGT, Eilat, Israel) in the morning and with fish eggs in the afternoon. ii) fish that were kept under a light/dark cycle of 6/18 hr for the same time period and weighing 1.44 ± 0.391 g (N = 32) and showing a HIS of 4.6 ± 0.3, with non-vitellogenic ovaries (group NV). This group was fed only with dry pelleted food. In order to show the reversibility of this condition, some of the non-vitellogenic females were placed in tanks under the regular 10 hr light/14 hr dark photoperiod cycle. Fish were kept at 25 ± 2°C and were fed twice a day, with shrimp nauplii (PGT, Eilat, Israel) in the morning and with fish eggs in the afternoon. Ovaries collected after three weeks from these females were in the vitellogenic stage.

Determination of sex and of developmental stages of ovaries, were done by microscopic examination of the gonads. Oocyte stages were determined according to [97]. The livers collected from all five groups were frozen instantly in liquid nitrogen and stored in -80°C until further use.

Measurements of E2 concentration in plasma was performed in similar designed separate experiments. Blood samples were collected using Micro-Hematocrit Tubes with Heparin (VWR, USA) from all tested groups and stored at -80°C. E2 concentrations were measured using Estradiol EIA Kit (Cayman, USA) according to the manufacture's protocol.

### RNA extraction

Total RNA was extracted from whole livers of zebrafish using Trizol reagent (GIBCO, USA) according to the manufacture's protocol followed by a clean-up and DNase treatement using RNeasy MiniElut Kit (Qiagene, Germany). After clean-up, 3 μl of the RNA samples were separated on 1.2% agarose gel to evaluate their quality and concentration. According to the gel picture, pooled samples from the livers of two fish were prepared for each group in Experiment 1 and of livers from eight fish for each group in the second experiment. Each RNA pool was quantified (A_260_) and assessed for purity (A_260_:A_280 _ratio) using Gene Quant (Amersham, UK) and by visual inspection of the 3 μg RNA separated on a denaturing gel.

### Zebrafish Oligonucleotide Microarray

Zebrafish microarrays were prepared by the Kimmel Cancer Center at Thomas Jefferson University (TJU), Philadelphia, USA. The microarray is a single color system based on zebrafish oligonucleotide library from Compugen/Sigma Genosys and consists of 16,399 oligonucleotide probes (65 nt), representing 16,288 unique gene clusters. In order to minimize non-specific binding, CodeLink slides with a special coating were used. Also β-actin internal controls were used to monitor the labeling and hybridization quality.

### Data Acquisition

Processed chips were scanned by using a Perkin Elmer ScanArray® XL 5000 Scanner, software version 3.1. Images were quantified by PerkinElmer (USA) Quant Array® Software 3.0. Quantization used the fixed circle method and outputs were total intensities. Microarray data were normalized across all arrays using quantile normalization of data in log base 2 scale [101]. This method corrects background noise and non-specific hybridization.

### Statistical analysis

Statistical analysis was performed using Significant Analysis of Microarray (SAM) software [102]. For the multiclass analysis a false discovery rate (FDR) of 0.1% was used. For paired t-test comparisons between the different groups a FDR of 0.03% was used. Correspondence Analysis (COA) was performed using MultiExperiment Viewer (MeV) version 4.1. Clustering analysis was performed using Coupled Two Way Clustering (CTWC) algorithm [103,105].

### cDNA synthesis and Real-Time PCR

Real-time PCR was performed using the same RNA samples used for microarray hybridization (n = 16). For cDNA synthesis, 4 μg of total RNA were mixed with 0.1 µg of oligo-d(T) (Promega, USA), 4 µl of Bio-RT 5× buffer, 2 µM dNTP mix (Promega, USA), 200 U of Bio-RT (Bio-lab, Israel) and H_2_O to reach a final volume of 20µl. After an incubation of 1 h at 37°C, 80 µl of H_2_O were added to the reaction. The PCR mixture consisted of 0.5 ul of cDNA sample, 70 nM of each primer (see Additional file 4) and 12.5 μl of SYBR Green master mix (ABgene, UK), in a final volume of 25 μl.

Amplification was carried out in a GenAmp 5700 thermocycler (PE Applied Biosystems, USA) and according to the manufacture's protocol. Amplification was performed in triplicates and the results were analyzed with REST-384 version 2 [106]. The relative expression of the 16 tested genes (see Additional file 4) was calculated using zebrafish *elongation factor 1 alpha *(*ef1a*) as a reference gene. The gene *ef1a *was recently shown to be a suitable reference gene for tissue analysis, developmental and E2 exposure studies of zebrafish [41,107].

### Data mining

The microarray annotations were updated using BlastX program against nr database of the GeneBank. Blast2GO software [108] was used for achieving GO annotations for the 2,523 differentially expressed genes found by SAM analysis (see Additional file 1 and Additional file 3). Combinations of gene lists were performed using Gene List Venn Diagrams software [109] (Fig. 5).

## Authors' contributions

LL designed and performed the experiments, the real-time PCR validation of the chips, the bioinformatic analyses of the data and was a major contributor in writing the manuscript. IP assisted in performing the experiments. EG and PF performed the microarray hybridization experiments of chips and participated in the writing of the manuscript. TH completed the normalization and SAM analysis of the chips data and participated in the writing of the manuscript. JB prepared the histological sections of the ovary. BLS contributed to the writing of the manuscript and provided the histological sections of zebrafish ovaries. EL conceived the study, participated in the design and coordination of the study, the analyses of data and was in charge of writing the manuscript. All the authors read and approved the final manuscript.

## Supplementary Material

Additional file 1 **Significantly different transcripts among the five tested groups. **List of 2, 523 genes corresponding to the significantly different transcripts found by SAM analysis. Click here for file

Additional file 2 **Detailed lists of genes that were found to be regulated in this study. **Detailed lists of gene accession numbers and gene names in groups 1–9 as they appear in Figures 6 and 7. Click here for file

Additional file 3 **Genes in selected GO terms. **This table represents SAM values and E2 regulation of genes in selected functions for the five tested groups. Click here for file

Additional file 4 **Primers used for chip validation. **List of primers used in real-time PCR for microarray validation. Click here for file

Additional file 5 **Validation of microarray results by real-time PCR. **The graphs in the figure indicate the correlation between the microarray results and real-time PCR results of 16 selected genes, represented by the R^2 ^value in each graph. The expression levels of the following 16 genes genes were normalized to *ef1a*: 1) *retinoic acid receptor alpha a *(*raraa*); 2) *estrogen receptor alpha *gene (*esr1*); 3) *retinol dehydrogenase *10 (*rdh10); *4) *retinol dehydrogenase *14 (*rdh14*); 5) *dehydrogenase/reductase (SDR family) member 10 *(*dhrs10*); 6) *stearoyl-desaturase *(sCd); 7) *fatty acid desaturase 2 *(*fads2*); 8) *alcohol dehydrogenase 5 (adh5); *9) *vitellogenin 1 (vtg1)*; 10) *vitellognin 3 *(*vtg3*); 11) *insulin-like growth factor 1 *(*igf1*); 12) *nothepsin *(*nots*); 13) *alcohol dehydrogenase 8b *(*adh8b*); 14) *cytochrome p450, family 1, subfamily a1 *(*cyp1a1*); 15) *cytochrome P450, family 2, subfamily K, polypeptide *6 (*cyp2k6*) and 16) *steroidogenic acute regulatory protein *(*star)*. Click here for file
