# Supplementary material for: Revealing genes associated with vitellogenesis in the liver of the zebrafish (Danio rerio) by transcriptome profiling
Source: BMC Genomics. 2009 Mar 31;10:141. doi: 10.1186/1471-2164-10-141 (PMC2678157; doi:10.1186/1471-2164-10-141)
Supplement: Additional file 3 — Genes in selected GO terms. This table represents SAM values and E2 regulation of genes in selected functions for the five tested groups. [file 1471-2164-10-141-S3.pdf]

Additional file 3: SAM values of genes in selected functions for the five tested groups: 2- and 4-month old females (Vit2 or Vit4, respectively), non- vitellogenic females (NV), E2-treated males, (E) and control males (M). Putatively E2-regulated genes are colored in yellow.

gene ID	Description	symbol	Score	Vit4	Vit2	NV	E	M
**GO:0042562**	**Hormone binding**							
AF349412	Estrogen receptor alpha	esr1	3.6	3.3	3.2	-4.9	3.2	-4.8
BG883146	Similar to kappa opioid receptor	oprk1	1.3	1.3	1.1	-2.2	1.2	-1.4
X70299	Nuclear receptor subfamily 2, group f 1	nr2f1	1.2	-0.6	0.9	2.0	-1.7	-0.6
AW153364	Similar to progesterone receptor membrane component 2	pgrmc2	1.1	1.7	0.5	-1.1	0.7	-1.7
AF220435	Steroidogenic acute regulatory protein (star)	star	1.1	-1.2	-1.3	2.1	-0.5	0.9
**GO:0015485**	**Cholesterol binding**							
BG884518	Similar to sterol o-acyltransferase 1	soat1	1.6	1.8	0.3	-1.7	2.0	-2.3
BI876786	Similar to steroid 5-alpha-reductase 2	srd5a3	1.1	1.3	-0.1	-1.4	1.6	-1.4
AF220435	Steroidogenic acute regulatory protein (star)	star	1.1	-1.2	-1.3	2.1	-0.5	0.9
**GO:0006629**	**Lipid metabolic process**							
AF395739	Lag1ceramide synthase 2	lass2	2.7	-0.1	-2.0	-2.5	6.1	-1.5
BE557760	Similar to cytochrome p450, family 8b	cyp8b	2.5	-0.3	2.2	2.1	-5.4	1.4
BI886664	Similar to palmitoyl-protein thioesterase 1	ppt1	2.4	-3.4	-1.2	3.7	-1.9	2.8
BI980068	Similar to l-3-hydroxyacyl-coenzyme a short chain	hadh	2.3	2.5	-2.6	-3.9	3.2	0.7
BM037477	Similar to solute carrier family 27 (fatty acid transporter) member 6	slc27a6	2.2	-2.8	-2.1	1.0	-0.2	4.1
AW170960	Similar to aldehyde dehydrogenase 8 member a1	adh8a1	2.2	0.1	-0.6	3.0	-4.4	1.8
BI891674	Similar to 15-hydroxyprostaglandin dehydrogenase	pgdh	2.1	-1.9	-0.8	4.2	-2.0	0.5
AF295372	Pyruvate carboxylase	pc	2.0	-1.4	-2.4	1.9	-1.7	3.6
AJ299412	High density lipoprotein-binding protein (vigilin)	hdlbp	1.9	4.3	-0.9	-0.9	-1.5	-1.1
AI877570	Similar to lysophospholipase isoform 2	pla2g4a	1.5	1.7	2.5	-0.6	-2.0	-1.5
AI601513	Similar to lipoprotein lipase	lpl	1.5	-1.0	-1.8	-0.4	3.1	0.0
BM185037	Similar to cytochrome p450, family 20, subfamily a1	cyp20a1	1.5	2.0	-0.8	-2.5	1.8	-0.6
AW078446	Similar to copine iii	cpne3	1.4	0.6	1.5	1.8	-2.1	-1.7
AW019835	Similar to cholesterol 7-alpha hydroxylase	cyp7a1	1.3	1.1	1.6	0.2	-2.4	-0.5
AW133873	Similar to hydroxy-delta-5-steroid3 beta-and steroid delta-isomerase 7	hsd3b7	1.3	-0.4	-0.3	2.3	-2.1	0.5
AW116322	Similar to acetyl-coenzyme a acyltransferase 2 (mitochondrial 3-oxoacyl-coenzyme a thiolase)	acaa2	1.3	-0.6	0.1	-2.5	1.6	1.4
AI477401	Similar to carnitine palmitoyltransferase ii	cpt2	1.3	0.3	-1.6	-1.8	1.5	1.6
AW422167	Similar to degenerative spermatocyte homolog 2 lipid desaturase	im:6909358	1.3	-0.8	0.0	2.9	-1.4	-0.6

AW116946	Similar to hydroxyacyl-coenzyme a dehydrogenase 3-ketoacyl-coenzyme a thiolase enoyl-coenzyme a hydratase (trifunctional protein) alpha subunit	hadha	1.3	-0.6	-0.9	-1.8	2.4	0.8
AW116229	Similar to hydroxy-delta-5-steroid3 beta- and steroid delta-isomerase 7	hsd3b7	1.3	-0.9	-0.6	2.2	-1.6	0.8
BM183259	Similar to macrophage migration inhibitory factor	mif	1.2	-0.9	0.0	1.8	-1.9	1.0
AW165251	Similar to membrane-bound transcription factor site 1	mbtps1	1.2	-0.8	1.4	1.0	-2.3	0.6
AW078386	Similar to udp-n-acetylglucosamine-2-epimerase n-acetylmannosamine kinase	gne	1.2	1.9	1.3	-1.4	0.0	-1.8
**GO:0008289**	**Lipid binding**							
AF254638	Vitellogenin 3, phosvitinless	vtg3	12.3	12.4	11.7	-16.0	10.8	-18.9
AF406784	Vitellogenin 1	vtg1	3.8	3.6	3.4	-2.0	2.4	-7.4
AF349412	Estrogen receptor alpha	esr1	3.6	3.3	3.2	-4.9	3.2	-4.8
AW018731	Similar to retinol binding protein cellular	rbp1	2.2	-3.5	-0.2	2.2	-2.0	3.5
AJ236882	Apolipoprotein e	apoeb	1.8	0.0	-0.1	2.4	-3.5	1.2
BI880006	Similar to human atp h+ mitochondrial f0 subunit c3 (subunit 9)	atp5g3	1.6	-0.9	-0.4	-1.4	3.4	-0.8
AI942949	Similar to solute carrier family 25 (carnitine acylcarnitine translocase) member 20	slc25a20	1.3	0.5	0.3	-2.8	1.7	0.2
AW153364	Similar to progesterone receptor membrane component 2	pgrmc2	1.1	1.7	0.5	-1.1	0.7	-1.7
AF220435	Steroidogenic acute regulatory protein (star)	star	1.1	-1.2	-1.3	2.1	-0.5	0.9
**GO:0006955**	**Immune response**							
BM181499	Similar to human guanylate binding protein interferon-67kda	gbp1	3.8	4.4	4.3	-6.3	1.3	-3.7
AY029577	X-box binding protein 1	xbp1	3.3	3.2	2.7	-5.5	2.9	-3.4
BG306390	Similar to interleukin enhancer binding factor 2	ilf2	1.9	-0.9	-1.2	-0.4	4.2	-1.8
BM037442	Similar to src family associated phosphoprotein 2	skap2	1.6	-1.7	-0.2	4.0	-1.6	-0.6
U08870	Major histocompatibility complex class ii dab gene	mhc2dab	1.6	-0.6	1.2	2.7	-2.3	-0.9
BG883146	Similar to kappa opioid receptor	oprk1	1.3	1.3	1.1	-2.2	1.2	-1.4
U08871	Mhc class ii beta chain	mhc2dfb	1.1	-1.2	0.1	2.5	-0.7	-0.6
**GO:0002376**	**immune system process**							
AF273220	Caspase 8	casp8	1.3	0.3	-1.4	2.4	-1.9	0.5
AI958611	Myosin, heavy chain 9, non-muscle, like-2	myh9l2	1.1	-0.8	-0.9	2.4	-1.0	0.4
AW134218	Glucose phosphate isomerase	gpi	1.2	-1.2	0.3	2.3	-1.6	0.2
BM037442	Similar to src family associated phosphoprotein 2	skap2	1.6	-1.7	-0.2	4.0	-1. 6	-0.6
U08870	Mhc class ii antigen	mhc2dab	1.6	-0.6	1.1	2.7	-2.3	-0.9
U08871	Mhc class ii beta chain	mhc2dfb	1.1	-1.2	0.1	2.5	-0.7	-0.6
U41081	Catenin beta1	ctnnb1	1.1	-1.0	-1.1	2.6	-0.3	-0.2

**GO:0007155**	**cell adhesion**							
AF203736	Cyclin-dependent kinase 5	cdk5	1.1	1.6	-1.4	1.5	-1.1	-0.6
AF260240	Claudin 7	cldn7	1.1	-0.8	-0.1	2.4	-0.8	-0.9
AJ011788	Claudin 7	cldn7	1.3	-0.6	-0.3	2.7	-1.2	-0.6
AF359426	Claudin 4	cldnb	1.8	-0.9	-1.1	4.4	-1.2	-1.3
AF359432	Claudin 3	cldnc	1.1	0.7	-0.5	2.0	-1.4	-0.8
AF428098	Cadherin 17	cdh17	1.5	-1.6	0	3.1	-0.8	-0.7
AI958611	Myosin, heavy chain 9, non-muscle, like-2	myh9l2	1.1	-0.9	-0.9	2.4	-1.0	0.4
AJ301602	Protein phosphatase 2 catalytic alpha isoform	zgc:56064	1.4	-0.9	-2.3	1.7	-0.3	1.9
BI892229	Cd9 antigen, like	cd9l	1.4	-0.7	0.1	2.9	-1.5	-0.9
U41081	Catenin beta1	ctnnb1	1.1	-1.0	-1.1	2.6	-0.3	-0.2
**GO:0003700**	**Transcription factor activity**							
AF349412	Estrogen receptor alpha	esr1	3.6	3.3	3.2	-4.9	3.2	-4.8
AY029577	X-box binding protein 1	xbp1	3.3	3.2	2.7	-5.5	2.9	-3.4
AF395739	Lag1ceramide synthase 2	lass2	2.7	-0.1	-2.0	-2.5	6.1	-1.5
BE017827	Similar to ccaat enhancer binding protein (cebp) delta	cebpd	2.3	1.3	1.0	1.2	-5.1	1.5
U40931	Paired box 9	pax9	2.2	2.0	2.8	-2.8	1.1	-3.1
AI883967	Similar to programmed cell death 11	pdcd11	2.1	0.9	0.7	-2.8	3.7	-2.5
AI588758	Similar to proliferation-associated 2g4	pa2g4b	2.1	0.2	-0.4	-2.4	4.3	-1.7
BI890771	Similar to prefoldin 1	pfdn1	2.1	0.0	-0.5	-2.4	4.4	-1.6
AI878713	Similar to ring finger protein 20	loc402838	1.8	-1.4	-2.7	-0.8	2.4	2.5
BI878967	Similar to apoptosis antagonizing transcription factor	aatf	1.7	-0.1	0.2	-1.4	3.5	-2.2
AY007990	Hairy and enhancer of split 6	her8a	1.7	3.5	-1.4	-1.7	-1.2	0.7
BI533952	Similar to phd-finger 5a	phf5a	1.6	0.6	1.1	-1.9	2.6	-2.4
BG729232	Similar to developmentally regulated gtp binding protein 1	drg1	1.6	1.2	-0.6	-1.9	2.8	-1.6
BI889426	Similar to hop homeobox	hop	1.5	-2.0	-0.2	2.2	-1.7	1.7
AF412832	Heat shock factor 2	hsf2	1.5	-1.7	0.0	1.6	-2.1	2.2
AY032595	Prospero-related homeobox 1	prox1	1.4	0.3	-1.5	0.8	-2.0	2.4
AJ005692	Signal transducer and activator of transcription isoform cra_a	stat1	1.4	-0.2	0.1	2.7	-2.4	-0.2
BI876457	Similar to sirtuin 6 (silent mating type information regulation homolog) 2	sirt6	1.4	-1.7	-0.1	2.6	-1.9	1.2
AF071260	Homeo box c12b	hoxc12b	1.3	2.1	-1.0	0.9	-2.2	0.2
BI867933	Similar to human taf9 rna polymerasetata box binding protein-associated factor	zgc:86811	1.3	-0.1	0.5	-1.0	2.4	-1.9
AF327373	Nuclear receptor sub family5 group a member 5	nr5a5	1.3	1.5	0.3	1.0	-2.6	-0.2
AA606218	Similar to polymeraseiii (dna directed) polypeptide k	zgc:92774	1.3	0.0	1.4	-1.0	1.6	-1.9
L03398	Retinoic acid alpha	raraa	1.2	0.0	-0.3	-1.2	2.6	-1.2
AF052245	Forkhead box a2	foxa3	1.2	2.4	-0.2	0.2	-2.0	-0.5
AF219989	Aryl hydrocarbon receptor nuclear translocator 2	arnt2	1.2	-0.1	-1.0	0.8	-1.8	2.0
X70299	Nuclear receptor subfamily 2, group f, member 1	nr2f1	1.2	-0.6	0.9	2.0	-1.7	-0.6
AF339838	Ets variant gene 6 (tel oncogene)	etv6	1.2	0.3	-0.4	0.4	-2.3	1.9

U41081	Catenin (cadherin-associated protein) beta 88kda	ctnnb1	1.2	-1.0	-1.1	2.6	-0.3	-0.2
AF342938	Peroxisome proliferator-activated receptor beta 2	pparb2	1.1	-0.9	0.1	1.5	-1.9	1.1
**GO:0000003**	**Reproduction**							
AF143493	T-complex 1	tcp1	1.4	1.2	-0.2	-2	2.3	-1
AF273739	Heat shock 10kda protein 1 (chaperonin 10)	hspe1	2.3	0.4	-1.1	-3	4.8	-1
AF364811	Novel proteine-cadherin	cdh1	1.6	-0.3	-1.6	2.5	-2	1.5
AF412832	Heat shock factor 2	hsf2	1.5	-1.7	0	1.6	-2	2.2
AI332057	Similar to tissue inhibitor of metalloproteinase 2	timp2	1.8	-1.9	-1.1	2.4	-2	2.7
AI601583	Similar to bystin-like	bysl	2.7	1.6	1.2	-4	4.2	-3
AI877506	Similar to nhp2 non-histone chromosome protein 2-like 1	zgc:56265	2.4	1.2	1.6	-2	3.4	-4
AW115780	Rna binding homolog 2	zgc:55287	1.4	-1.1	0.2	2	-2	1.2
AW116147	ATP-binding cassette, sub-family E (OABP), member 1	abce1	2.3	1.2	-0.1	-2	4.2	-3
BG883146	Similar to kappa opioid receptor	oprk1	1.3	1.3	1	-2	1.2	-1
BI876786	Similar to steroid 5-alpha-reductase 2	srd5a3	1.1	1.3	-0.1	-1	1.6	-1
BI890013	Similar to eukaryotic translation initiation factor 5a	zgc:77429	1.8	1.6	1.1	-2	1.9	-3
BI891674	Similar to 15-hydroxyprostaglandin dehydrogenase	pgdh	2.1	-1.9	-0.8	4.2	-2	0.5
**GO:0022414**	**Reproductive process**							
AF273739	Heat shock 10kda protein 1 (chaperonin 10)	hspe1	2.3	0.4	-1.1	-3	4.8	-1
AI601583	Similar to bystin-like	bysl	2.7	1.6	1.2	-4	4.2	-3
AI877506	Similar to nhp2 non-histone chromosome protein 2-like 1	zgc:56265	2.4	1.2	1.6	-2	3.4	-4
AW116147	ATP-binding cassette, sub-family E (OABP), member 1	abce1	2.3	1.2	-0.1	-2	4.2	-3
BG883146	Similar to kappa opioid receptor	oprk1	1.3	1.3	1	-2	1.2	-1
BI876786	Similar to steroid 5-alpha-reductase 2	srd5a3	1.1	1.3	-0.1	-1	1.6	-1
BI890013	Similar to eukaryotic translation initiation factor 5a	zgc:77429	1.8	1.6	1.1	-2	1.9	-3
