# Supplementary material for: Revealing genes associated with vitellogenesis in the liver of the zebrafish (Danio rerio) by transcriptome profiling
Source: BMC Genomics. 2009 Mar 31;10:141. doi: 10.1186/1471-2164-10-141 (PMC2678157; doi:10.1186/1471-2164-10-141)
Supplement: Additional file 4 — Primers used for chip validation. List of primers used in real-time PCR for microarray validation. [file 1471-2164-10-141-S4.pdf]

Additional file 4: List of primers used for microarray validation. The primers efficiency in real-time PCR was calculated according to a dilution curve prepared for each primer.

Gene	Primers	Product size	Efficiency in real-time PCR
adh5<br>AF399909	GTCCCAAAGCTGGTGAACGAC<br>AGCATGCATCAGGTCAAAGGC	111 bp	E=1.99
adh8b<br>AY309075	GTTCGGAGCCACAGATTTCC<br>GTTGCCCACACACTCCAGAGA	113 bp	E=2
vtg1<br>NM_170767	ACTACCAACTGGCTGCTTAC<br>ACCATCGGCACAGATCTTC	100 bp	E=2
vtg3<br>AF254638	CAGATGGCTTTATCGGCGTGAC<br>CACGGCAGGCCCATTGAAAC	112 bp	E=2
efia<br>L23807	AATCGGTGGTGCTGGCAAGGTC<br>AAGAACTCGCCGCAACCTTTG	101 bp	E=2
cyp2k6<br>AF283813	TGAGTGGGAAACGCCTGA<br>GCCAAACTCTCTCCAATGCA	126 bp	E=2
raraa<br>L03398	ATGGATGACGCTGAGACGGGAC<br>GTGTGGCCTTCTGTTCCTCACG	147 bp	E=1.8
cyp1a<br>AF057713	GCAGGTTTTGACACTATCAGT<br>GACTCCAGAAGCGGCAG	160 bp	E=2
igf1<br>AF268051	CCTCGAGATGTATTGTGCG<br>TACAGGAAGAGTGGCTATGC	125 bp	E=1.8
esr1<br>AF349412	TAGTTGACGCATGTGCCA<br>TAGGAGCGCTCACTCTGATC	123 bp	E=1.9
rdh10<br>BI890056	TTTGCCGTTTGATGCCATTGTG<br>TCGTCGACTCCTTGCGTTGAGC	92 bp	E=2
rdh14<br>BF718032	TCCTGGCCTGCAGAGACATGAG<br>CTGCAAAGAGGCCAGATCCAGC	110 bp	E=2
dhrs10<br>BI888372	CAGGGTATTGGCAGGGCTTTCG<br>TGCGCCACATCCTCAGCCTTTC	91 bp	E=2
scd<br>BF717695	GGATCACCGCGTGCATCACAAG<br>TTCGAACCAGCAGCCAGCCAAC	101 bp	E=1.9
fads2<br>AF309556	CAGCGGACACCTCAACTT<br>AGCCAGAGCTCGCCAGA	188 bp	E=1.8
star<br>AF220435	ACCTGTTTTCTGGCTGGGATG<br>GGGTCCATTCTCAGCCCTTAC	81 bp	E=1.9
