# Supplementary figures and images for: Revealing genes associated with vitellogenesis in the liver of the zebrafish (Danio rerio) by transcriptome profiling
Source: BMC Genomics. 2009 Mar 31;10:141. doi: 10.1186/1471-2164-10-141 (PMC2678157; doi:10.1186/1471-2164-10-141)

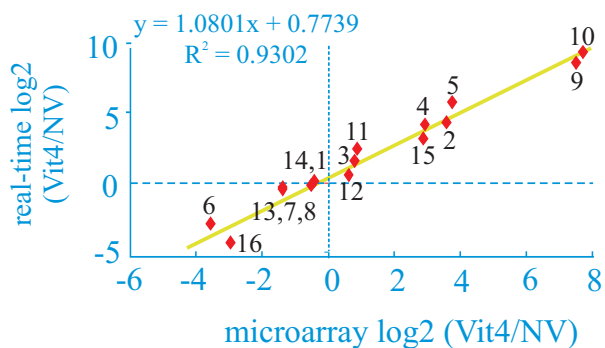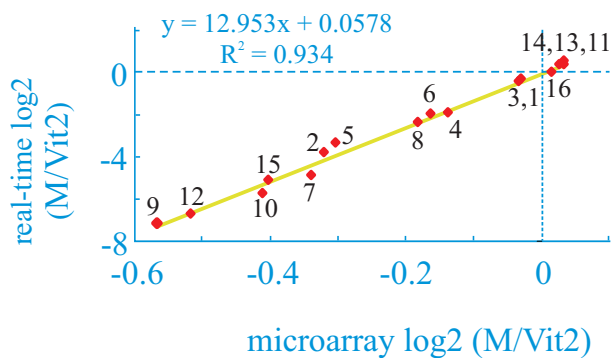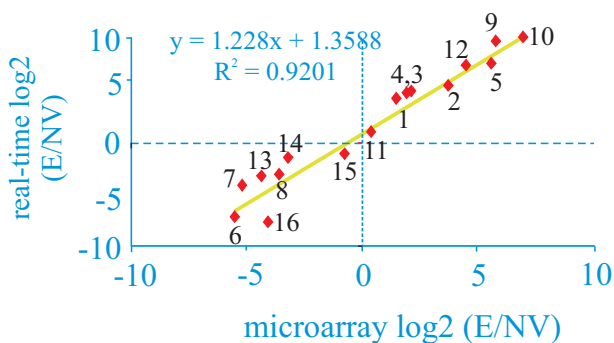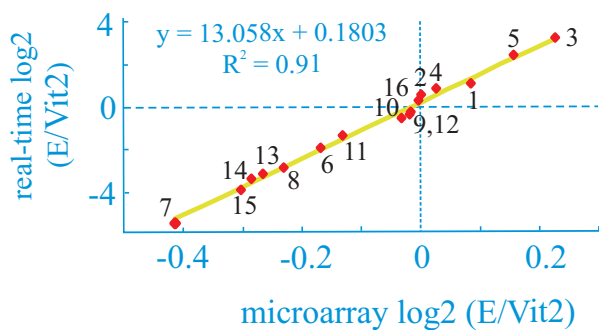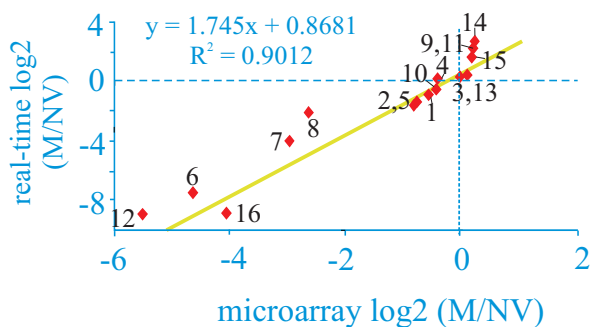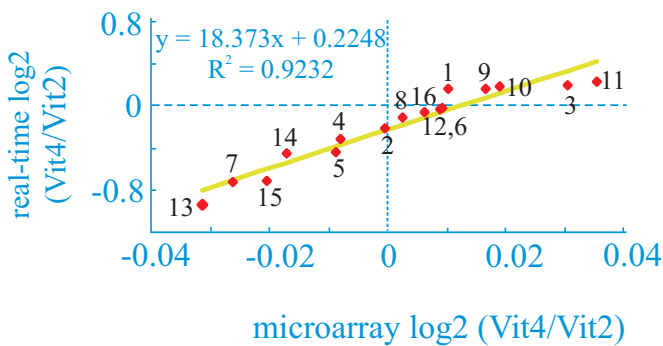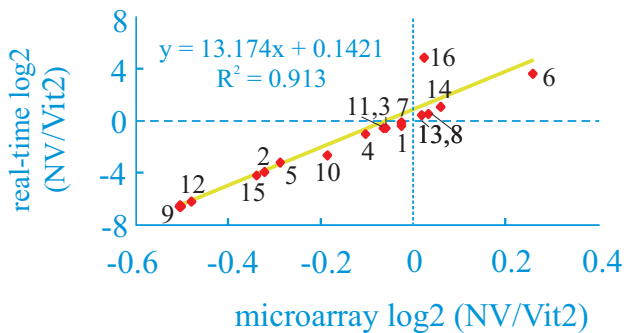

Supplement: Additional file 5 — Validation of microarray results by real-time PCR. The graphs in the figure indicate the correlation between the microarray results and real-time PCR results of 16 selected genes, represented by the R2 value in each graph. The expression levels of the following 16 genes genes were normalized to ef1a: 1) retinoic acid receptor alpha a (raraa); 2) estrogen receptor alpha gene (esr1); 3) retinol dehydrogenase 10 (rdh10); 4) retinol dehydrogenase 14 (rdh14); 5) dehydrogenase/reductase (SDR family) member 10 (dhrs10); 6) stearoyl-desaturase (sCd); 7) fatty acid desaturase 2 (fads2); 8) alcohol dehydrogenase 5 (adh5); 9) vitellogenin 1 (vtg1); 10) vitellognin 3 (vtg3); 11) insulin-like growth factor 1 (igf1); 12) nothepsin (nots); 13) alcohol dehydrogenase 8b (adh8b); 14) cytochrome p450, family 1, subfamily a1 (cyp1a1); 15) cytochrome P450, family 2, subfamily K, polypeptide 6 (cyp2k6) and 16) steroidogenic acute regulatory protein (star). [file 1471-2164-10-141-S5.pdf]
